# Are Adolescents Engaged in the Problematic Use of Social Networking Sites More Involved in Peer Aggression and Victimization?

**DOI:** 10.3389/fpsyg.2018.00801

**Published:** 2018-05-29

**Authors:** Belén Martínez-Ferrer, David Moreno, Gonzalo Musitu

**Affiliations:** Department of Education and Social Psychology, Pablo de Olavide University, Sevilla, Spain

**Keywords:** online social networking sites, problematic use of Internet, peer aggression, peer victimization, adolescence

## Abstract

The problematic use of social networking sites is becoming a major public health concern. Previous research has found that adolescents who engage in a problematic use of social networking sites are likely to show maladjustment problems. However, little is known about its links with peer aggression and victimization. The main goal of this study was to analyze the relationship between problematic use of online social networking sites, peer aggression –overt vs. relational and reactive vs. instrumental–, and peer victimization –overt physical and verbal, and relational–, taking into account gender and age (in early and mid-adolescence). Participants were selected using randomized cluster sampling considering school and class as clusters. A battery of instruments was applied to 1,952 adolescents' secondary students from Spain (Andalusia) (50.4% boys), aged 11 to 16 (*M* = 14.07, *SD* = 1.39). Results showed that girls and 14–16 adolescents were more involved in a problematic use of online social networking sites. Furthermore, adolescents with high problematic use of online social networking sites were more involved in overt—reactive and instrumental—and relational—reactive and instrumental—aggressive behaviors, and self-reported higher levels of overt—physical and verbal—and relational victimization. Even though boys indicated higher levels of all types of victimization, girls with high problematic use of online social networking sites scored the highest on relational victimization. Relating to age, early adolescents (aged 11–14) with higher problematic use of online social networking sites reported the highest levels of overt verbal and relational victimization. Overall, results suggested the co-occurrence of problematic use of online social networking sites, peer aggression and victimization. In addition, results showed the influence that gender and age had on peer victimization. This study highlights the continuity between offline and online domains with regard to maladjustment problems in adolescence.

## Introduction

The use of Internet and Online Social Networking Sites (SNS) is an increasingly popular leisure activity in many countries around the world (Kuss and Griffiths, 2011). It is part of today's lifestyle, especially in adolescence and youth (Johansson and Götestam, 2004; Devine and Lloyd, 2012). SNS constitute one of the most frequent communication tools among adolescents to establish and maintain new interpersonal relationships, allowing them to participate in social groups (Hearn and Foth, 2007). In fact, SNS have replaced other virtual communication tools, such as e-mail and text messages, as well as non-virtual ones (García et al., 2012). In the online domain, characterized by immediacy, anonymity and globalization, adolescents build their identity, strengthen existing social relationships and create new social bonds (Muñoz-Rivas et al., 2003). One of the most attractive features of SNS is precisely the possibility to be an active agent in the process of social interaction beyond geographical proximity (Echeburúa and de Corral, 2010).

However, the use of the Internet and the SNS is not without risks. Ease of access to the Internet as well as the immediacy and social interaction capabilities of these tools also seem to increase the risk of developing problematic Internet use (PIU) and, in particular, problematic use of SNS (O'Reilly, 1996; Wellman and Gulia, 1999; Preece, 2000), which in some cases can even be classified as addictive (Smahel et al., 2008; Fioravanti et al., 2012; Gómez Salgado et al., 2014). The concept of Internet addiction, however, has not been fully developed, and research in this area remains incipient and fragmented (Yang and Tung, 2007), and no consensus criteria have been established to differentiate between excessive and problematic use of the Internet or dependence.

This plurality of terms in scientific literature reveals that there are multiple conceptual approaches to this problem and a lack of a diagnostic category, such as Internet addiction (Fernández-Villa et al., 2015). All this hinders reaching a consensual definition of terms such as excessive use of the Internet, PIU, and dependency, that consider different conceptual nuances. Despite these limitations, we can say that both Internet addiction and the problematic or excessive use of this tool refer to an individual's inability to control his or her use of the Internet with negative effects deriving from this lack of regulation, such as psychological distress and functional impairment (Echeburúa and de Corral, 2010; Fernández-Villa et al., 2015). Meanwhile, these conceptual difficulties have been reproduced in the specific field of SNS. Andreassen and Pallesen (2014) define addiction to SNS as an excessive concern to connect to these interaction spaces dedicating such a large amount of time that other areas such as social activities, studies/work, interpersonal relationships, psychological health, and well-being are affected. However, just as with the concept of Internet addiction, other terms have been chosen in scientific literature, such as excessive or problematic use of the SNS, to avoid the diagnostic or clinical connotation proper to the term “addiction.”

The problematic use of the Internet and SNS usually affects populations who are vulnerable because of their age, such as adolescents (Griffiths and Wood, 2000; Pallanti et al., 2006; Puerta Cortés and Carbonell, 2014). Previous studies have indicated that 2 to 15% of adolescents use the Internet and SNS in an excessive and problematic way (Park et al., 2008; Durkee et al., 2012; Sasmaz et al., 2013). In Spain, 96.5% of children under 15 years usually access the Internet (Spanish Statistical Office, 2012), 11.5% of adolescents connect between 3–5 h a day and 5.5% use the Internet more than 5 h a week, a percentage that rises in late adolescence (Catalina et al., 2014). In addition, ~85% of adolescents are registered in at least one SNS (Bringué and Sádaba, 2009; Rial et al., 2014) and average age of initiation to Facebook has dropped to 12 years (García et al., 2012). Likewise, it has been estimated that between 3.7 and 10% of adolescents make excessive or problematic use of these virtual tools (Muñoz-Rivas et al., 2010; Carbonell et al., 2012). This high level of usage of SNS, at problematic frequency rates in adolescence, can be attributed to the fact that these virtual spaces constitute a scenario to explore and develop multiple identities away from the supervision of parents or other figures of formal authority (Mazzoni and Iannone, 2014; Andreassen, 2015).

Regarding gender, available empirical evidence offers inconclusive results. It has been observed that boys tend to have a higher PIU than women (McKenna and Bargh, 2000; Morahan-Martin and Schumacher, 2000; Anderson, 2001; Schumacher and Morahan-Martin, 2001; Durkee et al., 2012). However, in other studies, no significant differences were found on PIU or Internet dependency according to gender (Kim and Davis, 2009). One significant aspect allowing to examine gender differences in more detail is how these technologies are used. Thus, data obtained from different studies show that boys spend more hours per week using e-mail, online games and web page visits, while girls use chats and social networks more (Muñoz-Rivas et al., 2003; Andreassen et al., 2012). In fact, the problematic use of SNS has been found to be greater in girls, especially in young and adolescent girls (Ryan et al., 2014; Andreassen, 2015), probably because girls tend to engage more in activities involving social interaction than boys (Andreassen et al., 2012; Kuss et al., 2014; Van Deursen et al., 2015).

The consequences of the problematic use of the Internet and SNS in adolescents have aroused growing concerns in the scientific community and in society (Blaszczynski, 2006; Echeburúa et al., 2009). There is abundant empirical evidence that the PIU and the problematic use of SNS is associated with greater manifestations of adjustment problems in adolescence such as psychological distress (Andreassen and Pallesen, 2014), anxiety (Chabrol et al., 2017), depressive symptoms (Morrison and Gore, 2010; Cao et al., 2011; Pedrero et al., 2012), loneliness, and social isolation (Young and Rogers, 1998; Satici et al., 2014), and emotional problems (Caplan, 2010; Marengo et al., 2018). Furthermore, in a meta-analysis, Marino et al. (2018) found that the problematic use of Facebook was related to greater psychological distress, anxiety and depression, and low life satisfaction. The problematic use of the Internet and SNS has also been related to externalizing problems in adolescence (Rodríguez and Fernández, 2014), with higher levels of impulsivity (Young, 1999; Echeburúa et al., 2009), anger and hostility (Ko et al., 2008; Xiuqin et al., 2010; Adalier and Balkan, 2012; Carli et al., 2013), and poor impulse control (Grüsser et al., 2007). In the same way, adolescents who used Internet more frequently were found to be more likely to participate in aggressive and hostile behaviors (Ko et al., 2008; Xiuqin et al., 2010; Adalier and Balkan, 2012; Carli et al., 2013; Mak et al., 2014). More specifically, adolescents who spent more time connected to SNS showed to be more frequently involved in peer aggression behaviors such as bullying (Ko et al., 2012) and cyberbullying (Erdur-Baker, 2010; Navarro et al., 2013; Giménez et al., 2015; Kircabum and Bastug, 2017).

**Figure 1:**
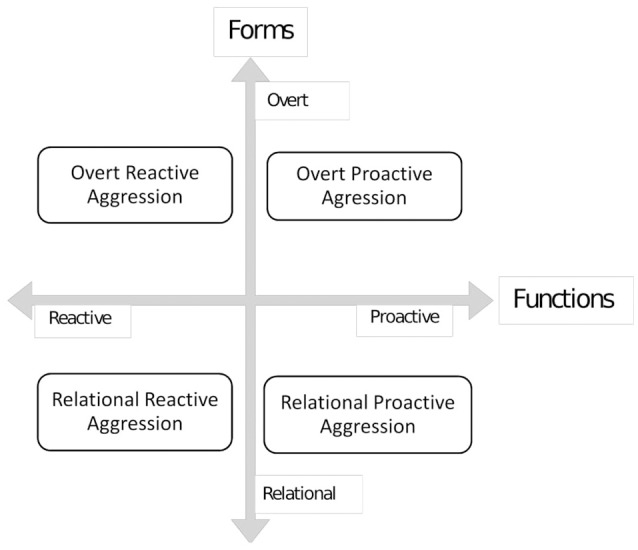
Subtypes of aggressive behavior according to Little's classification.

One limitation of these studies is that aggression is analyzed globally. However, not all aggressive behavior is due to high impulsivity. It is important to distinguish between different manifestations of peer aggression in adolescence, to be able to explain the variety of its causes and associated correlates (e.g. Crick and Dodge, 1996). In this line, Little's taxonomy (Little et al., 2003; see Card and Little, 2006; Card et al., 2008) is based on two axes: the forms (“whats”-overt vs. relational aggression) and functions (“whys” -reactive vs. proactive) (See Figure 1). Forms refer to the type of behavior intending to harm the victim: aggressive conduct may involve a direct confrontation with others (e.g., intimidating, pushing, hitting, threatening, or insulting-overt aggression) or, on the contrary, may imply damaging the social reputation or social status of victims and isolate them from their friends, going so far as to use their group of peers (e.g., social exclusion, social rejection, or spreading rumors; Xie et al., 2002; Little et al., 2003; Juvonen and Graham, 2014) With regard to functions, i.e., the aggression's purpose, this type of conduct has been found to be due possibly to reacting to real or perceived damage -reactive aggression-, or a means to obtain a desired result -proactive aggression- (Coie and Dodge, 1998). While reactive aggression is fundamentally emotional and impulsive (Card and Little, 2006), proactive aggression implies an intentional behavior directed toward fulfilling a desired goal and, therefore, depends on an evaluation of the consequences (Fontaine and Dodge, 2006; Frey et al., 2017).

By combining these two axes (forms and functions) four dimensions or subtypes of aggressive behavior are obtained: overt -reactive and proactive-, and relational –reactive, and proactive. This classification has been validated ecologically and these dimensions, although interrelated, have shown important differences (see Murray-Close et al., 2016). In addition, the conceptualization of forms and functions as subcategories contributes to a better understanding of the motives and socio-cognitive mechanisms underlying this behavior (Juvonen and Graham, 2014). Regarding victimization, defined as the experience of being subjected to physical, verbal and psychological aggression perpetrated by peers (Graham, 2006), adolescents with greater use of SNS have shown a greater probability of experiencing cyber-victimization experiences (Navarro et al., 2013; Giménez et al., 2015). However, it seems that no studies have yet analyzed victimization experiences in non-virtual contexts and the problematic use of the Internet and SNS.

### The current study

The problematic use of the Internet and SNS and their associated correlates is still at a very early stage. Most studies have generally analyzed the relationship between Internet dysfunctional use, PIU, psychological distress, internalizing, and externalizing problems. Findings show that adolescents with greater problematic use of the Internet and SNS are more likely to engage in behaviors that involve aggression, hostility and impulsivity. In fact, it has been suggested that adolescents displaying problematic use of Internet and SNS have more impulse control difficulties, which would explain not only their dysfunctional usage of Internet and SNS, but also their greater involvement in aggressive behavior among peers. However, important questions remain, since not all manifestations of aggression imply high impulsivity and poor control of impulses.

Therefore, the present study has two main goals. First, the present study aimed to analyze the relationships between the problematic use of SNS and offline peer aggression, considering the forms (overt vs. relational) and the functions (reactive vs. proactive) of aggressive behavior, and as a function of gender and age. Second, despite the empirical evidence on the excessive use of Facebook and other SNS and cyber-victimization, no studies have been found that explore the link between the problematic use of the Internet or SNS and peer victimization. Since the virtual and the non-virtual settings are interrelated (Castells, 2001; Chóliz and Marco, 2012), the present study also aimed to analyze the relationship between offline peer victimization (overt and relational) and the problematic use of SNS, considering gender and age. This study may contribute to specifically advancing the knowledge about the link between several subtypes of offline peer aggression and victimization and the problematic use of social online sites that aim to strengthen social relationship and create new ones. On this basis, we posed the following hypothesis:

H1: It was expected that adolescents who show a problematic use of SNS were more involved in all the forms -overt and relational- and functions -proactive and reactive- of offline peer aggression.
H2: It was expected that the relationship between problematic use of SNS and offline peer aggression was different as a function of gender and age.
H3: It was expected to find that adolescents with a problematic use of SNS reported higher levels of both overt and relational offline victimization.
H4: It was expected that the relationship between problematic use of SNS and offline peer victimization was different as a function of gender and age.

## Materials and methods

### Participants

The study was ex post facto transversal and descriptive. In this study, the initial sample consisted of 2,083 adolescents of both sexes aged 12–16 years. A total of 81 adolescents were excluded for the following reasons: not having attended one of the three phases of the study, normally due to illness (56%), mistakes in the answers (e.g., more than one answer was given for the same item on at least one scale) (28%), difficulties in understanding Spanish (foreign students) (12%), and students who voluntarily abandoned the study or who responded systematically in the same way to all scales (4%). Finally, 1,952 adolescents (50.4% boys) participated, aged 11–16 years (*M* = 14.07, *SD* = 1.39), enrolled in nine compulsory secondary education centers (ESO) in Andalusia (Spain). The participants attended public (56.4%) and semi-private (43.5%) schools. Two age groups corresponding to different stages in adolescence were established: early adolescence (11–13 years old, 37.3%) and middle adolescence (14–16 years old, 62.7%).

For the sample, the average of missing data was 2.1%, and never above 5% for an individual measure. The low level of missingness meant that it was not likely to bias the results, thus the estimations were accurate to the expected values on the population (Graham, 2009). Missing values by scales or subscales were processed using the regression imputation method. In this method, rows in the data matrix are presumed to constitute a random sample of a normal multivariate population. Univariate outliers were detected via the exploration of standardized scores. Following the criteria provided by Hair et al. (2016), atypical values were those whose standardized scores had an absolute value above 4. For multivariate detection, Mahalanobis distance was computed. A multivariate outlier is identified if the associated probability at a Mahalanobis distance is 0.001 or less (Tabachnick and Fidell, 2007).

### Instruments

#### Peer aggression

**Figure 2:**
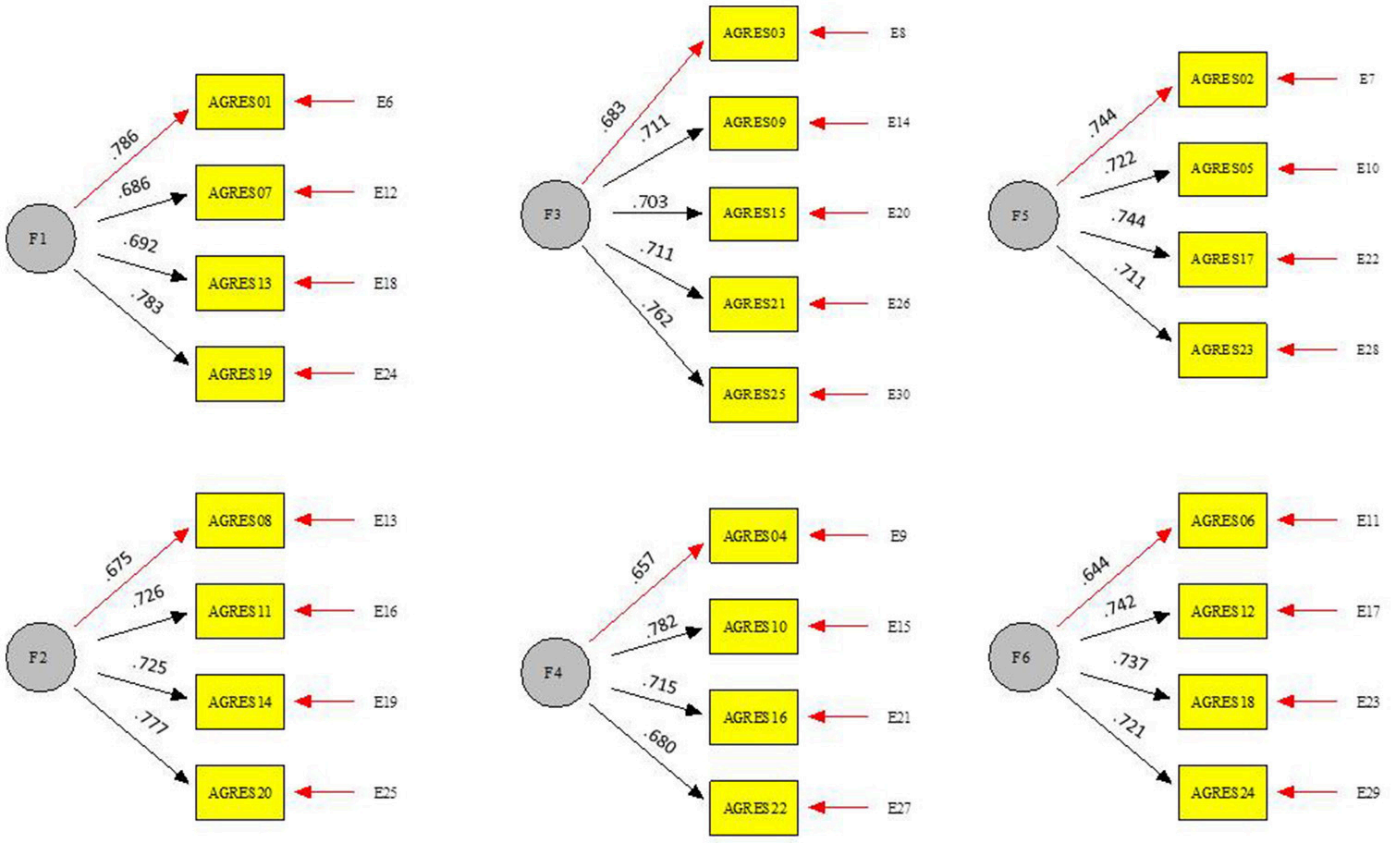
Peer aggression scale. ^***^p < 0.001. Correlation F1-F2, *r* = 0.855^***^;Correlation F1-F3, *r* = 0.822^***^; Correlation F1-F4, *r* = 0.853^***^;Correlation F1-F5, *r* = 0.720^***^; Correlation F1-F5, *r* = 0.720^***^; Correlation F1-F6, *r* = 0.704^***^; Correlation F2-F3, *r* = 0.769^***^; Correlation F2-F4, *r* = 0.587^***^; Correlation F2-F5, *r* = 0.685^***^; Correlation F2-F6, *r* = 0.678^***^; Correlation F3-F4, *r* = 0.783^***^; Correlation F3-F5, *r* = 0.692^***^; Correlation F3-F6, *r* = 0.941^***^; Correlation F4-F5, *r* = 0.810^***^; Correlation F4-F6, *r* = 0.887^***^; Correlation F5-F6, *r* = 0.743^***^. CFA fit index: [S-Bχ^2^ = 527.5385, *df* = 241, *p* < 0.001, CFI = 0.910, RMSEA = 0.026(0.023, 0.030)].

The Aggressive Behavior Scale was used (Little et al., 2003). This scale consists of 35 items with four response options (1 = never and 4 = always), which measure participation in aggressive behavior toward peer groups. For the present study, the subscales of overt reactive aggression (four items, Cronbach's alpha [α] = 0.76, composite reliability [ρ_c_] = 0.75, Ω = 0.82, average variance extracted [AVE] = 53%) (e.g., “When someone harms or hurts me, I hit him”), overt proactive aggression (four items, α = 0.78, ρ_c_ = 0.78, Ω = 0.84, AVE = 53%) (e.g., “I threaten others to get what I want”), relational reactive aggression (four items, α = 0.77, ρ_c_ = 0.77, Ω = 0.80, AVE = 51%) (e.g., “When someone makes me angry, I treat him/her with indifference or I stop talking to him/her”) and relational proactive aggression (four items, α = 0.72, ρ_c_ = 0.75, Ω = 0.80, AVE = 51%) (e.g., “to get what I want, I disparage others”). The confirmatory factor analysis [CFA] performed showed the measurement model had a good fit with the data [SBχ2 = 527.5385, *df* = 241, *p* < 0.001, CFI = 0.915, RMSEA = 0.026, I.C. 90 (0.023, 0.030)]. Taking into consideration the following values, the overall reliability of the scale was acceptable (α = 0.95, ρ_c_ = 0.95, Ω = 0.96, AVE = 52%) (See Figure 2).

#### Peer victimization

**Figure 3:**
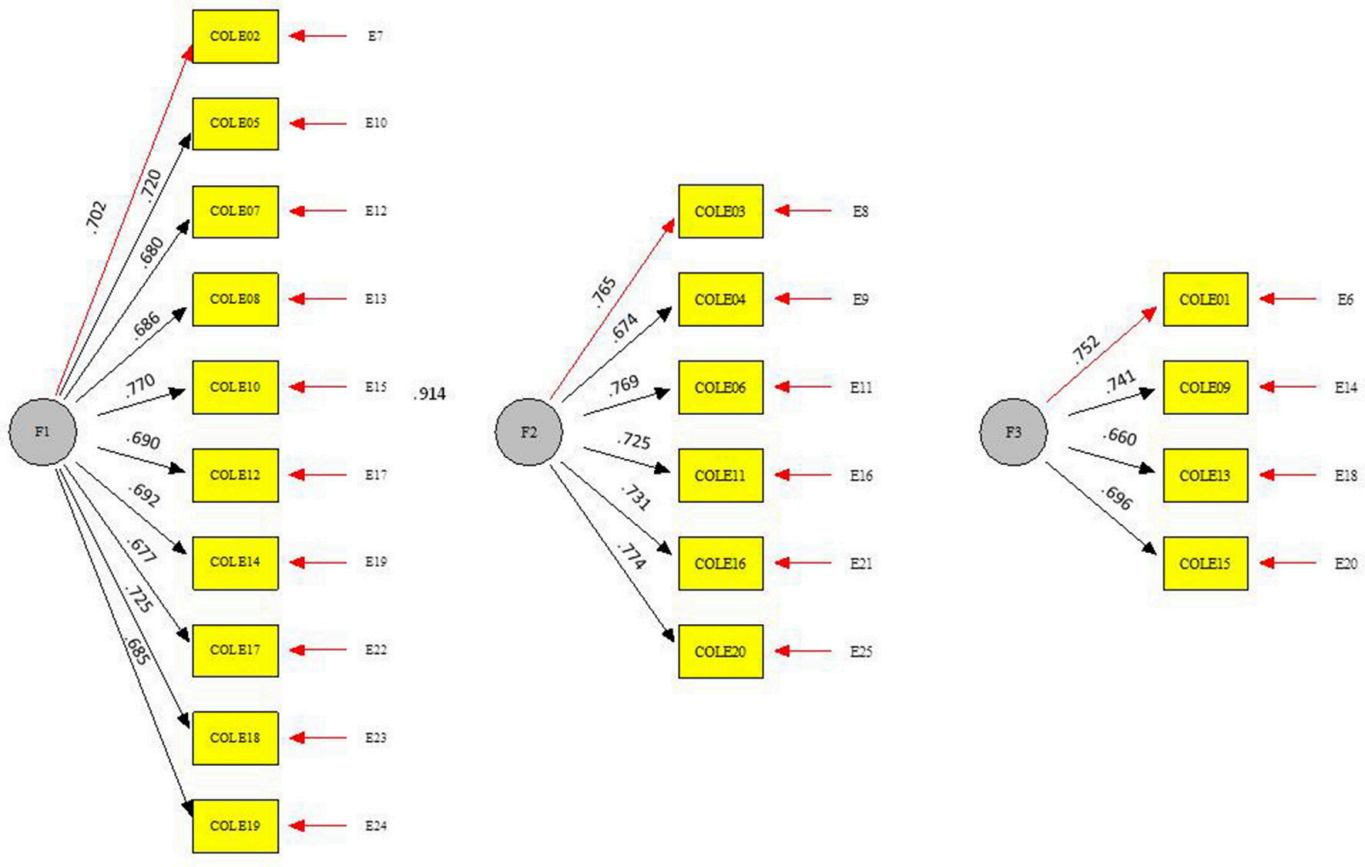
Peer victimization scale. ^***^p < 0.001. Correlation F1-F2, *r* = 0.968^***^;Correlation F1-F3, *r* = 0.756^***^;Correlation F2-F3, *r* = 0.885^***^; CFA fit index: [S-Bχ^2^ = 421.1204, *df* = 158, *p* < 0.001, CFI = 0.930, RMSEA = 0.038(0.034, 0.043)].

The School Victimization Scale (Mynard and Joseph, 2000) is composed of 20 items that refer to peer victimization situations, with four response options (1 = never and 4 = always). The scale has three factors: relational victimization (ten items, α = 0.89 ρ_c_ = 0.88, Ω = 0.91, AVE = 55%) (e.g., “a peer spread rumors about me and criticized me behind my back”), verbal overt victimization (six items, α = 0.83 ρ_c_ = 0.83, Ω = 0.88, AVE = 55%) (e.g., “A peer insulted me”), and physical overt victimization (four items, α = 0.71 ρ_c_ = 0.74, Ω = 0.80, AVE = 15%) (e.g., “a peer struck or hit me to really harm me”). The CFA performed showed a good fit of the measurement model to the data [SB χ2 = 421.1204, df = 158, *p* < 0.001, CFI = 0.930, RMSEA = 0.038 (0.034, 0.039)]. The overall reliability of the scale was acceptable (α = 0.92, ρ_c_ = 0.94, Ω = 0.96, AVE = 51%) (See Figure 3).

#### Problematic use of SNS

**Figure 4:**
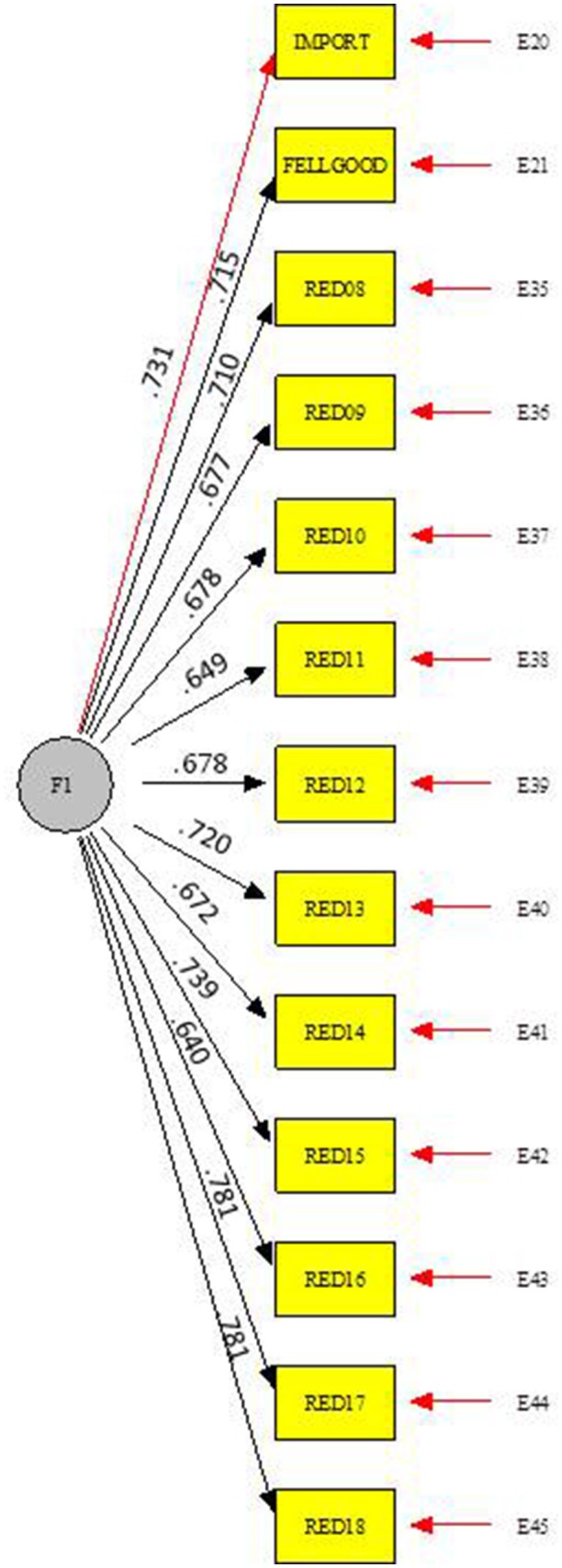
Problematic use of SNS. CFA fit index: [S-Bχ^2^ = 141.4920, gl = 57, *p* < 0.001, CFI = 0.964, RMSEA = 0.036(0.029, 0.043)].

A scale was administered to assess problematic use of SNS in adolescence. This scale is composed of 13 items with a response range of 1 (never) to 4 (always) it measures the problematic use of SNS (e.g., “I need to be connected to my social networks continuously”). The CFA showed a good fit of the proposed measurement model [SB χ2 = 141.4920, *p* < 0.001, df = 57; CFI = 0.96, RMSEA = 0.036, I.C. 90 (0.029, 0.043)]. The scale showed an acceptable reliability (α = 0.87, ρ_c_ = 0.90, Ω = 0.93, AVE = 51%) (See Figure 4).

### Procedure

Schools were selected randomly in Andalusia (Spain). First, we contacted the principals of the selected educational centers, explained the research project to them and asked for their agreement. After this initial contact, we asked for an informative seminar to be held addressed to teachers as well as members of administration to explain the research objectives and to request parents' authorizations. Next, a letter describing the study was sent to parents, requesting them to indicate in writing whether they accepted their child's participation in the study (only 1.5 % of the parents refused). Participants anonymously and voluntarily filled out the scales during regular classroom hours (45 min). Trained researchers administered the instruments to the adolescents during the school day, informing them always that their participation in the study was voluntary and anonymous. The study met the ethical requirements proper to research with human beings, respecting the fundamental principles included in the Declaration of Helsinki.

### Data analysis

Confirmatory Factorial Analysis (CFA) using EQS (6.1) (Bentler, 2006) was performed to examine the construct validity of the scales. We used the Maximum Likelihood estimation method and the Satorra-Bentler scaled chi-square test for non-normal data (Yuan et al., 2000). We also calculated the Comparative Fit Index (CFI) and the Tucker-Lewis Index (TLI), where acceptable or good fit is indicated when values above 0.90 or 0.95, respectively, are obtained. Root Mean Square Error of Approximation (RMSEA) values of 0.05 or less indicate good model fit (Hu and Bentler, 1998). Cronbach's alpha was also calculated. Factor loadings were assessed for statistical significance at the *p* < 0.01 level. As for validity, Composite Reliability, AVM, and McDonald Omega were calculated. Composite reliability values above 0.60, AVM values above 0.50, and McDonald Omega values above 0.70 indicate an acceptable convergent validity (Hair et al., 1998; Gefen et al., 2000; Lance et al., 2006).

Statistical analyses were performed using the statistical package SPSS, version 23, and missing values were handled using the regression imputation method (Allison, 2001). First, groups of problematic SNS use were formed. For this, three groups of adolescents were identified: low problematic use, with scores less than or equal to the first quartile, *N* = 467 (23.9%), average problematic use, with scores in the interquartile range, *N* = 961 (49.2 %) and high problematic use, with scores equal to or greater than the third quartile, *N* = 524 (26.8%). A correlational analysis was also performed to analyze relationships between variables; descriptive analyses were calculated to examine how sociodemographic gender and age group variables were distributed in the variables under study. A multifactorial MANOVA was carried out to achieve the goals of the study.

Next, a MANOVA was carried out to analyze differences in overt -proactive and reactive- and relational -proactive and reactive- aggression and overt - physical and verbal- and relational victimization, depending on the problematic use of SNS, gender and age. The factorial design was 3 (problematic use of low, medium and high SNS) by 2 (gender, boy or girl) by 2 (age group: from 11 to 13 years and from 14 to 16 years), to examine possible interaction effects. Next, univariate tests (ANOVAS) were performed to examine differences in variables that were statistically significant and the Bonferroni *post-hoc* test was applied. (α = 0.05).

## Results

### Descriptive analyses

Before performing the multivariate analysis, a zero–order correlation analysis was computed for all the variables. As Table 1 shows, problematic use of SNS was significantly and positively related to both relational and overt victimization—physical and verbal, and to both overt and relational aggression—reactive and instrumental. Boys scored higher on all dimensions of peer aggression and overt victimization—physical and verbal. However, girls scored higher on relational victimization and problematic use of SNS. Moreover, adolescents aged 14–16 years scored higher on overt reactive aggression, relational reactive aggression, and problematic use of SNS, whereas adolescents from 11 to 13 years scored higher on overt physical and verbal victimization.

**Table 1:** Pearson correlations among the variables in the study, mean, and standard deviations.

	**1**	**2**	**3**	**4**	**5**	**6**	**7**	**8**	**9**	**10**
1. Gender^a^	1									
2. Age^b^	0.01	1								
3. ORA	−0.36^**^	0.05^*^	1							
4. OIA	−0.11^**^	0.03	0.40^**^	1						
5. RRA	−0.00	0.09^**^	0.31^**^	0.31^**^	1					
6. RIA	−0.07^**^	−0.03	0.26^**^	0.61^**^	0.40^**^	1				
7. OPV	−0.16^**^	−0.06^*^	0.16^**^	0.15^**^	0.03^**^	0.11^**^	1			
8. OVV	−0.05^*^	−0.07^**^	0.13^**^	0.18^**^	0.13^**^	0.16^**^	0.60^**^	1		
9. RV	0.08^**^	0.03	0.05^*^	0.17^**^	0.18^**^	0.20^**^	0.51^**^	0.75^**^	1	
10. PSNSU	0.08^**^	0.16^**^	0.22^**^	0.23^**^	0.23^**^	0.22^**^	0.12^**^	0.17^**^	0.21^**^	1
M (*SD*)	1.50 (0.50)	1.41 (0.49)	1.59 (0.55)	1.13 (0.24)	1.77 (0.48)	1.19 (0.30)	1.19 (0.29)	1.60 (0.46)	1.46 (0.43)	1.83 (0.46)

*M, Mean; SD, Standard Deviation; ORA, Overt Reactive Aggression; OIA, Overt Instrumental Agression; RRA, Relational Reactive Aggression; RIA, Relational Instrumental Aggression; OPV, Overt Physical Victimization; OVV, Overt Verbal Victimization; RV, Relational Victimization; PSNSU, Problematic Social Networking Sites Use*.

a *Gender: 1 = boys, 2 = girls*.

b *Group Age: 1 = 11–13, 2 = 14–16*.

* *p < 0.05*;

** *p < 0.01*.

Next, we examined whether the groups were similar in terms of demographic variables. As shown in Table 2, significant differences relating to gender and group age were found between groups. Regarding gender, results showed significant differences between groups [χ^2^ (2) = 16.45, *p* < 0.001]. Girls were overrepresented in the group with high problematic use of SNS (*N* = 294, 30.3%); whereas boys were overrepresented in the low problematic use group (*N* = 265, 27%), and the percentages of boys and girls were similar in average problematic use group (N boys = 488, 49.6%, N girls = 473, 48.8%). As for age, results also showed significant differences among groups [χ^2^ (2) = 45.80, *p* < 0.01]. Adolescents aged 14 to 16 were overrepresented on average (N [14-16] = 608, 63.3%), as well as in high problematic use of SNS (N [14-16] = 377, 71.9%), while adolescents from both groups were similar in low use of SNS (N [11-13] = 228, 48.8%, N [14-16] = 239, 51.2%). This data coincides with results obtained from previous studies in the Spanish context (Muñoz-Rivas et al., 2010; see Carbonell et al., 2012).

**Table 2:** Sociodemographic variables.

**Variables**		**Problematic use SNS**			
	**Total sample**	**Low (*N* = 467)**	**Medium (*N* = 961)**	**High (*N* = 524)**	**χ^2^**
Gender					χ^2^(2) = 16.45^***^
Boys	983(50.4%)	265(56.7%)	488(50.8%)	230(43.9%)	
Girls	969(49.6%)	202(43.3%)	473(49.2%)	294(56.1%)	
Group age					χ^2^(2) = 45.80^***^
[11–13]	728(37.3%)	228(48.8%)	353(36.7%)	147(28.1%)	
[14–16]	1, 224(62.7%)	239(51.2%)	608(63.3%)	377(71.9%)	

*χ^2^, Chi-square*.

*** *p < 0.001*.

### Multivariate analysis

In the calculated MANOVA, statistically significant differences were found in main effects of gender variables [Λ = 0.822, *F*_(7, 1934)_ = 64.41, *p* < 0.001, η2p = 0.178], age [Λ = 0.982, *F*_(7, 1934)_ = 5.07, *p* < 0.001, η2p = 0.018] and problematic use of the SNS [Λ = 0.881, *F*_(14, 3868)_ = 18.11, *p* < 0.001, η2p = 0.062]. A statistically significant interaction effect was also obtained between problematic use of SNS and gender [Λ = 0.983, *F*_(14, 3868)_ = 2.37, *p* < 0.01, η2p = 0.009] and problematic use of SNS and age [Λ = 0.986, *F*_(14, 3868)_ = 1.93, *p* < 0.05, η2p = 0.007].

Regarding sociodemographic variables, in the ANOVA, statistically significant differences were obtained with respect to gender in the variables overt reactive, overt proactive, and overt instrumental aggression. As shown in Table 3, boys showed higher scores than girls in overt aggression (reactive and proactive), proactive relational aggression, and overt victimization (physical and verbal), while girls scored higher in relational victimization. In relation to age, results of the ANOVA indicated that adolescents aged 14–16 years had higher scores in relational reactive aggression and lower scores in overt victimization (physical and verbal), compared to adolescents between 11 and 13 years old.

**Table 3:** Means, standard deviations, and differences on peer aggression and peer victimization by gender, age, and problematic use of SNS.

	**Gender**				**Age**				**Problematic use SNS**				
	**Boys**	**Girls**	***F*_(1, 1952)_**	**$\eta_{\text{p}}^{2}$**	**11–13**	**14–16**	***F*_(1, 1952)_**	**$\eta_{\text{p}}^{2}$**	**Low**	**Medium**	**High**	***F*_(2, 1952)_**	**$\eta_{\text{p}}^{2}$**
ORA	1.79(0.59)	1.39(0.42)	284.80^***^	0.128	1.55(0.55)	1.61(0.55)	0.726	0.000	1.41^c^(0.49)	1.60^b^(0.52)	1.74^a^(0.61)	69.82^***^	0.067
OIA	1.16(0.27)	1.10(0.21)	26.27^***^	0.013	1.12(0.25)	1.14(0.25)	0.077	0.000	1.07^c^(0.21)	1.12^b^(0.21)	1.21^a^(0.31)	37.54^***^	0.037
RRA	1.77(0.48)	1.77(0.48)	0.018	0.000	1.71(0.48)	1.80(0.47)	8.32^**^	0.004	1.59^c^(0.45)	1.78^b^(0.44)	1.90^a^(0.51)	44.44^***^	0.044
RIA	1.21(0.32)	1.17(0.29)	11.84^**^	0.006	1.18(0.31)	1.20(0.30)	0.313	0.000	1.13^c^(0.27)	1.77^b^(0.27)	1.28^a^(0.37)	31.90^***^	0.032
OPV	1.23(0.32)	1.14(0.25)	59.10^***^	0.030	1.21(0.31)	1.17(0.27)	11.89^**^	0.006	1.16^b^(0.29)	1.18^b^(0.25)	1.23^a^(0.34)	15.25^***^	0.015
OVV	1.62(0.47)	1.57(0.46)	7.26^**^	0.004	1.64(0.49)	1.57(0.45)	23.16^***^	0.012	1.52^b^(0.49)	1.57^b^(0.41)	1.71^a^(0.52)	31.45^***^	0.031
RV	1.42(0.41)	1.49(0.44)	9.89^**^	0.005	1.48(0.45)	1.44(0.41)	13.07^***^	0.007	1.36^c^(0.43)	1.43^b^(0.36)	1.59^a^(0.50)	41.97^***^	0.041

*ORA, Overt Reactive Aggression; OIA, Overt Instrumental Agression; RRA, Relational Reactive Aggression; RIA, Relational Instrumental Aggression; OPV, Overt Physical Victimization; OVV, Overt Verbal Victimization; RV, Relational Victimization*.

** *p < 0.01*,

*** *p < 0.001, a > b > c*.

Concerning the problematic use of SNS, results of the ANOVA showed significant differences in all dimensions analyzed (see Table 3). The Bonferroni tests (α = 0.05) indicated that adolescents with the highest problematic use of the SNS obtained the highest scores in overt and relational aggression (both proactive and reactive, overt victimization -physical and verbal) and relational victimization, compared to adolescents with medium and low use. In addition, adolescents with an average use of SNS, compared to the low-use group, reported higher scores for overt and relational aggression (both proactive and reactive, and relational victimization), and similar scores to the low use group for overt victimization (physical and verbal).

#### Analysis of interactions

**Figure 5:**
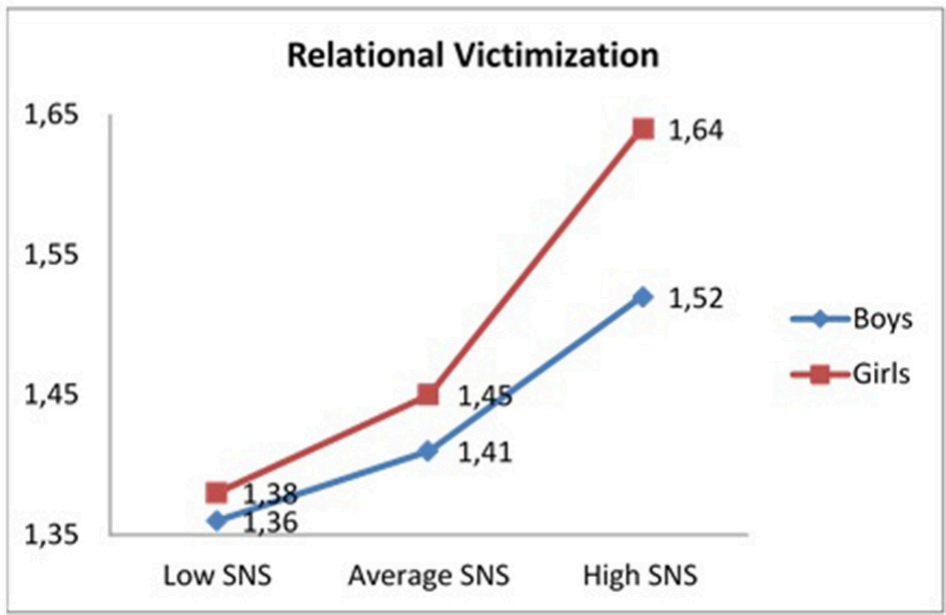
Interaction effect problematic use SNS-low, average, and high-,gender, and relational victimization.

A statistically significant interaction effect was obtained between problematic use of SNS, and gender and relational victimization, *F*_(5, 1946)_ = 17.75, *p* < 0.001, η^2^p = 0.044. As seen in Table 4, the results of the *post-hoc* contrasts performed with the Bonferroni test (α = 0.05) indicated that girls, particularly those with greater problematic use of SNS, obtained the highest scores in relational victimization, compared to the remaining groups (see Figure 5).

**Table 4:** Mean, Standard Deviation (SD,) and *post-hoc* comparisons between problematic use of SNS, gender, age, overt verbal victimization, and relational victimization.

		**Problematic use SNS**			
	**Gender**	**Low**	**Average**	**High**	***Post hoc* comparisons**
RV^1^	Boys	1.36 (0.43)^a^	1.41 (0.35)^b^	1.52 (0.48)^c^	c > a, b, d
	Girls	1.38 (0.42)^d^	1.45 (0.38)^e^	1.64 (0.52)^f^	f > a, b, c, d, e
	**Age**	**Low**	**Average**	**High**	***Post hoc*** **comparisons**
OVV^2^	11-13	1.55(0.50)^a^	1.59 (0.40)^b^	1.79 (0.57)^c^	c > a, b, d, e, f
	14-16	1.55 (0.51)^d^	1.56 (0.42)^e^	1.55 (0.44)^f^	
RV^3^	11-13	1.39(0.45)^a^	1.43(0.35)^b^	1.64(0.55)^c^	c > a, b, d, e, f
	14-16	1.41 (0.45)^d^	1.44 (0.37)^e^	1.45 (0.41)^f^	

*RV, Relational Victimization; OVV, Overt Verbal Victimization. α = 0.05*.

1 *F _(5, 1946)_ = 17.75, p < 0.001, η^2^p = 0.044*;

2 *F _(5, 1946)_ = 12.07, p < 0.001, η^2^p = 0.030*;

3 *F_(5, 1946)_ = 2.42, p < 0.001, η^2^p = 0.034*.

**Figure 6:**
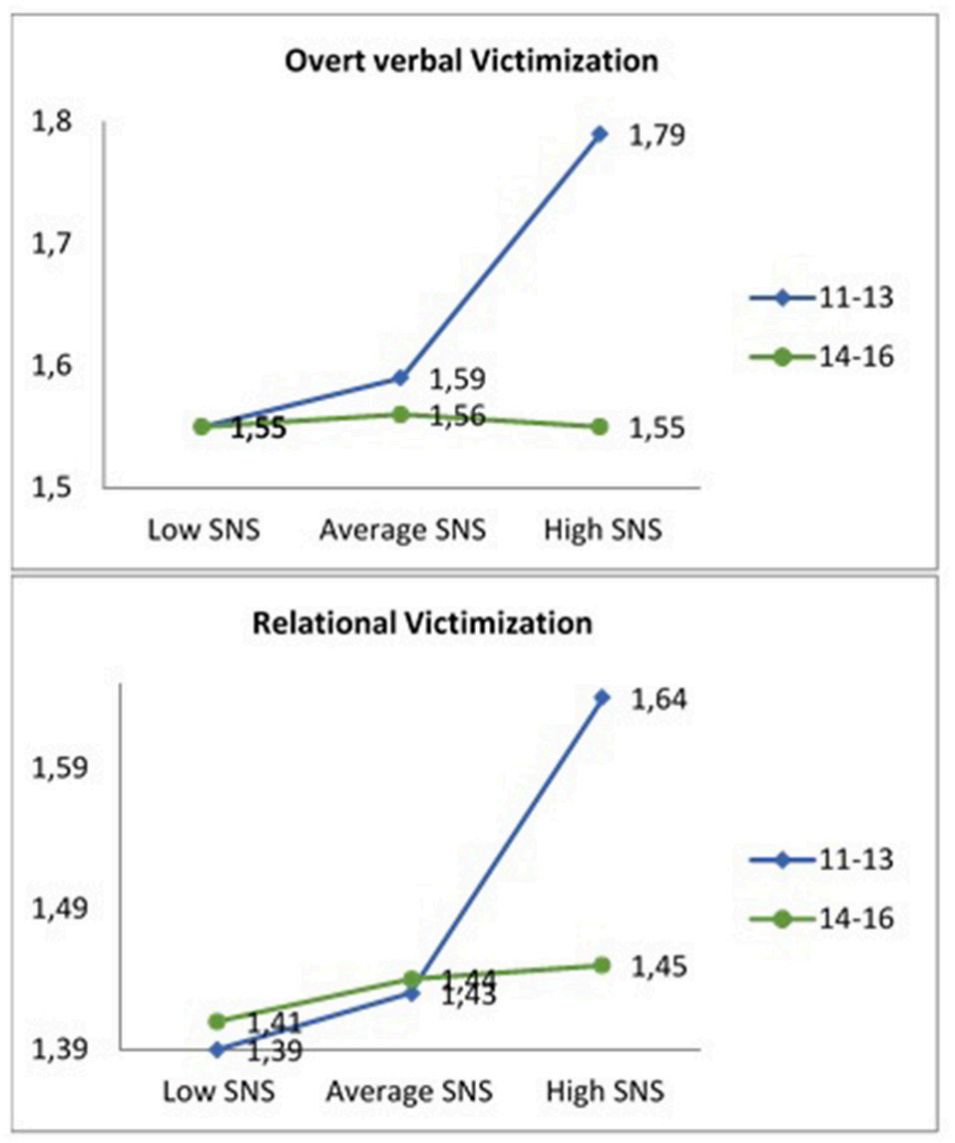
Interaction effect problematic use SNS-low, average, and high-,age,overt verbal victimization, and relational victimization.

A statistically significant interaction effect was also obtained between problematic use of SNS, age and overt verbal victimization, *F*_(5, 1946)_ = 12.07, *p* < 0.001, η^2^p = 0.030. Results of the *post-hoc* contrasts performed with the Bonferroni test showed that adolescents aged 11–13 with a higher problematic use of SNS reported higher overt verbal victimization, compared to the remaining groups (see Table 4 and Figure 6). This trend was also observed in the interaction between problematic use of SNS, age and relational victimization, *F*_(5, 1946)_ = 2.42, *p* < 0.001, η^2^p = 0.034. As shown in Table 4 and Figure 6, 11–13-year-old adolescents with the most problematic use of SNS obtained the highest scores in relational victimization compared to the rest of the groups.

## Discussion

The general goal of the present study was to analyze existing relationships between the problematic use of SNS, peer aggression and victimization in adolescents, taking into account gender and age. In addition, the forms (overt vs. relational) and the functions (proactive vs. reactive) of aggressive behavior were considered to evaluate peer aggression. Forms of victimization were considered as well. Overall, results confirmed that the problematic use of SNS was associated with greater involvement in all dimensions of analyzed peer aggression and with higher levels of overt (physical and verbal) victimization and relational victimization. Likewise, gender and age were shown to have an important role in the relationship between problematic use of SNS and relational victimization and verbal expression.

The first specific objective of this study was to examine the relationship between peer aggression and the problematic use of SNS, by gender and age. We hypothesized that adolescents with a problematic use of SNS would be more involved in all the subtypes of aggressive behavior measured. As expected, results showed that adolescents with greater problematic use showed a greater involvement in overt and relational aggression, with both a reactive and a proactive purpose. These results are compatible with those obtained in previous studies in which the PIU has been linked to Internet addiction with greater hostility and greater expression of aggressive behaviors in adolescents (Ko et al., 2008, 2012; Xiuqin et al., 2010; Adalier and Balkan, 2012; Carli et al., 2013).

However, results of the present study show a deeper analysis of these relationships: the greater the problematic use of SNS, the greater the manifestation of peer aggression, in its different forms -manifest and relational- and functions -reactive and instrumental. Our findings could be explained because high engagement in aggressive conducts, both proactive and reactive, can be attributed to the fact that both types of aggression are interrelated (Poulin and Boivin, 2000; see Kempes et al., 2005).

Previous research has linked PIU and Internet dependence to difficulties in controlling impulses (Grüsser et al., 2007; Echeburúa et al., 2009; Kim and Davis, 2009), which may explain the relationship between the problematic use of SNS and reactive aggression. However, proactive aggression, unlike reactive aggression, is a more elaborate, planned and, therefore, less impulsive behavior: it is thus linked less to these difficulties in impulse control. In fact, impulsivity and anger have shown to be key factors in distinguishing between both types of aggression (Raine et al., 2006). Conversely, proactive (or instrumental) aggression has been associated with a reduced ability to delay gratifications, so that the possible benefits are valued by the aggressors, regardless of the damage they may cause to the victims (Kempes et al., 2005; Ramírez and Andreu, 2006; Crespo-Ramos et al., 2017). Both traits, impulsivity and difficulties on delaying rewards are also linked to problematic use of Internet and SNS. Additional research, though, is needed to test whether these characteristics might mediate the association between Problematic use of SNS and peer aggression. In relation to the role of gender and age, results indicate that boys participate more frequently than girls in all analyzed dimensions of aggression, except in the relational reactive dimension, where equivalent scores were found. Regarding age, the only differences were in reactive relational aggression, where adolescents aged 11–13 were found to be more frequently involved in these behaviors.

Regarding the second specific objective, results from previous studies indicate that adolescents with greater use of SNS report greater cyber-victimization (Navarro et al., 2013; Giménez et al., 2015). The findings of this study indicated that, as hypothesized, the problematic use of SNS was associated with the experience of overt -physical and verbal- and relational victimization. In addition, gender and age seemed to play an important role in understanding these relationships. This conclusion was drawn from analyzing the interaction between problematic use of SNS, gender, and age with respect to victimization. Thus, in relation to gender, results indicated that boys and girls showed a similar degree of relational peer victimization in the problem use groups of the lower and middle SNS. However, in the high problematic use group, adolescents reported higher levels of relational victimization. We believe that this result is highly revealing, insofar that it is evident that adolescents with problematic use of SNS also present other adjustment problems that differ according to gender.

As results obtained in the univariate analyses show, boys were subject to relational victimization more frequently than girls, while reporting a higher problematic use of SNS. This result is compatible with that obtained by other authors (Bringué and Sádaba, 2009; Griffiths et al., 2014; Ryan et al., 2014; Andreassen, 2015). Girls seemed to use Internet tools more often than boys, primarily for communication, strengthening friendships and social interaction (Muñoz-Rivas et al., 2003; Espinar Ruiz and González Río, 2009; Andreassen et al., 2012). Nevertheless, when these variables were taken into account, girls with the most problematic use of SNS clearly reported greater relational victimization, mainly aimed at excluding adolescents from the peer group and eroding their social life.

In adolescence, SNS represent a platform to establish new relationships and to strengthen existing ones, whether friendly or romantic. Girls, more than boys, seem to find the online domain a safer and more protected space to initiate friendship and affective relationships (Subrahmanyam and Greenfield, 2008). However, a greater predisposition to online communication seems to entail a greater risk of suffering negative consequences derived from the problematic use of the Internet and SNS (Oberst et al., 2017). Results of the present study suggest that these risks are especially high in girls showing problems of relational victimization. Thus, it is possible that in a situation of victimization, girls participate more frequently in SNS and in online communication activities to strengthen existing friendship relationships and to create new links that minimize the adverse effects of the experience of victimization: this, in turn, may make these adolescents more vulnerable to developing a problematic use of SNS. This finding can be considered highly significant and these relationships should be analyzed more in-depth in future research.

Regarding the interaction between problematic use of SNS and age, results showed that adolescents aged 11–13 years old -early adolescence- who used the SNS in a problematic way, reported greater verbal and relational verbal victimization, compared to the rest of the groups. Previous research has shown that approximately between 3.7% and 10% of adolescents used the Internet in a problematic way. However, no data has been found regarding the problematic use of SNS at different stages of adolescence. These results are compatible with studies carried out in Spain, that found that 85% of adolescents were registered in at least one SNS (Bringué and Sádaba, 2009; García et al., 2012; Rial et al., 2014). The findings of the present study indicated that adolescents aged 14–16 years showed greater problems with the use of SNS. However, it was adolescents aged 11–13 years with high problematic use who reported greater overt verbal and relational victimization. These results could be explained by the fact that the highest prevalence of peer victimization occurs in early adolescence (Martínez-Ferrer et al., 2011; Povedano et al., 2012). In addition, at this developmental stage, they already have access to the SNS and, therefore, they can make a greater exploratory use of these tools, which could lead to a high and problematic use of the SNS. For adolescents, one of the most inviting aspects of these networks is that they enable building support networks, searching information and social support beyond geographical proximity, though often reduced to the virtual domain (Muñoz-Rivas et al., 2003; Echeburúa and de Corral, 2010). It is possible that the SNS constitute, in turn, a support-seeking tool for adolescents victimized by their peer group,

## Limitations

Despite its positive aspects, this study also presents some limitations. Positive aspects include the novelty of the field, which lacks almost completely in empirical evidence, in particular on the link between problematic use of SNS and peer victimization. In this sense, results of the present study contribute to scientific knowledge in this field. Another main contribution is related to the interaction between gender and problematic use of SNS. This result sheds light on the need to incorporate a gender perspective in future research focused on the continuity of the processes of aggression and victimization in the online and offline domains. Among its limitations, the cross-sectional nature of the study did not allow establishing causal relationships between studied variables, so longitudinal studies should test these relationships more in depth. In this sense, future longitudinal studies could examine these relationships in more detail. In addition, all sources were self-reports, leading to possible biases, especially in sensitive topics such as aggression and the problematic use of SNS. In this sense, the use of online surveys could minimize the aforementioned biases because adolescents might feel their identity more protected. Moreover, outliers and missing values could be easily identified. Furthermore, findings suggest that as pointed out by (Livingstone and Smith, 2014; Smith and Livingstone, 2017), offline and online continuity exists, so vulnerable adolescents in one area may also be vulnerable in the other, while those who take risks in one domain may also take them in others. Future research may find it revealing to incorporate measures of cyber-aggression and of cyber-victimization.

## Conclusions

Despite its limitations, the results of this study allow addressing the extent of the problematic use of SNS in depth and connecting the offline and online settings. Many studies have found that the problematic use of Internet and SNS is associated with other existing adjustment problems such us internalizing symptoms and lower levels of life satisfaction (Caplan, 2010; Marengo et al., 2018). This study contributes to a better understanding of adolescents with a problematic use of SNS and its links to peer aggression and peer victimization. Findings of the present study are particularly important given that research on SNS and adjustment problems in offline settings is scarce. The constellation of adjustment problems is interrelated, so adolescents with more problems deriving from the use of these social networks are more frequently involved in -overt and relational- aggression and victimization. In addition, in girls and adolescents aged 11–13 years, the problematic use of SNS is linked to greater victimization suggesting social influences. These findings reveal the need for educational programs on Internet and SNS good practices so that adolescents develop a healthy use of these communication tools. Such programs could also help detect violence and peer victimization problems and, therefore, lead to initiatives to improve harmonious coexistence in the community. Moreover, at the public health level, identifying the problematic use of SNS among adolescents in the early stages could potentially lead to identifying improvements in preventing peer aggression and fostering positive social climates.

## Ethics statement

The present study was conducted in accordance with the 1964 Helsinki declaration and its later amendments or comparable ethical standards, with the approval of the management board of schools, the educational inspection services, and the Childhood Observatory of the Regional Government of Andalusia (Spain). All participants gave written informed consent. The required authorizations from the education authorities, the schools, and the children's families were obtained.

## Author contributions

BM-F, DM, and GM had participated in the intellectual content, the analysis of data, and the writing of the work. BM-F, DM, and GM had reviewed the final version of the work and they approve it for publication.

### Conflict of interest statement

The authors declare that the research was conducted in the absence of any commercial or financial relationships that could be construed as a potential conflict of interest.
